# Reproductive and endocrine effects of artemisinin, piperaquine, and artemisinin-piperaquine combination in rats

**DOI:** 10.1186/s12906-022-03739-2

**Published:** 2022-10-13

**Authors:** Xiaobo Li, Yueming Yuan, Yingyi Chen, Li Ru, Zheng Yuan, Zhiyong Xu, Qin Xu, Jianping Song, Guoming Li, Changsheng Deng

**Affiliations:** 1 Artemisinin Research Center, Guangzhou University of Chinese Medicine, Guangzhou, China; 2 Sci-tech Industrial Park, Guangzhou University of Chinese Medicine, Guangzhou, China; 3 The First Affiliated Hospital of Guangzhou University of Chinese Medicine, Guangzhou, China

**Keywords:** Artemisinin, Piperaquine, Reproduction, Endocrine, Rats

## Abstract

**Background:**

The WHO recommends artemisinin-based combination regimens for uncomplicated *Plasmodium falciparum* malaria. One such combination is artemisinin-piperaquine tablets (ATQ). ATQ has outstanding advantages in anti-malarial, such as good efficacy, fewer side effects, easy promotion and application in deprived regions. However, the data about the reproductive and endocrine toxicity of ATQ remains insufficient. Thus, we assessed the potential effects of ATQ and its individual components artemisinin (ART) and piperaquine (PQ) on the reproductive and endocrine systems in Wistar rats.

**Methods:**

The unfertilized female rats were intragastric administrated with ATQ (20, 40, and 80 mg/kg), PQ (15, 30, and 60 mg/kg), ART (2.5, 5, and 10 mg/kg), or water (control) for 14 days, respectively. The estrous cycle and serum levels of estradiol (E2), follicle-stimulating hormone (FSH), luteinizing hormone (LH), prolactin (PRL), prostaglandin (PG), and adrenocorticotropic hormone (ACTH) were determined. The weights of the kidney, adrenal gland, uterus, and ovaries were measured. The histopathological examinations of the adrenal gland, ovary, uterus, and mammary gland were performed.

**Results:**

Compared with the control group, there were no significant differences in the examined items of female rats in the ART groups, including general observation, estrous cycle, hormonal level, organ weight, and histopathological examination. The estrous cycle of female rats was disrupted within 4–7 days after ATQ or PQ administration, and then in a persistent dioestrus phase. At the end of administration, ATQ and PQ at three doses induced decreased PG, increased ACTH, increased adrenal weight and size, and pathological lesions in the adrenal gland and ovary, including vasodilation and hyperemia in the adrenal cortex and medulla as well as hyperplasia and vacuolar degeneration, ovarian corpus luteum surface hyperemia, numerous but small corpus luteum, and disordered follicle development. But the serum levels of E2, FSH, LH, and PRL did not change obviously. These adverse effects in ATQ or PQ treated rats could not completely disappear after 21 days of recovery.

**Conclusion:**

Based on the results of this study, ART had no obvious reproductive and endocrine effects on female rats, while ATQ and PQ caused adrenal hyperplasia, increased ACTH, decreased PG, blocked estrus, corpus luteum surface hyperemia, and disrupted follicle development in female rats. These events suggest that ATQ and PQ may interfere with the female reproductive and endocrine systems, potentially reducing fertility.

## Background

Malaria is an infectious disease that seriously endangers human health. It is caused by parasites and spreads to people through the bites of infected female Anopheles mosquitoes. According to the World Health Organization (WHO) world malaria report [1], there are approximately 241 million cases of malaria worldwide, resulting in 627,000 deaths in 2020, most of which concern children under the age of 5. Malaria is prevalent in Africa, Southeast Asia, and the Mediterranean region, and Africa is the most seriously affected region. The WHO recommends that the best available treatment, particularly for *Plasmodium falciparum* malaria, is artemisinin-based combination therapy (ACT), which combines artemisinin or its derivatives with a partner drug. Artemisinin and its derivatives are highly potent drugs for multi-drug resistant *Plasmodium falciparum* treatment. These compounds cause a swift parasite reduction, have broad parasite stage specificity, and are effective against all *Plasmodium spp.* in humans. Due to the short half-life of artemisinin and its derivatives, combination therapies of an artemisinin-based compound and long-acting antimalarial drugs are gaining importance.

One of these combination therapies is the fixed-dose artemisinin-piperaquine tablets (ATQ) initially developed by Chinese scientists. The specification for ATQ is each tablet contains 375 mg PQ and 62.5 mg ART. The clinical usage and dosage of ATQ are as follows: oral administration, the course of treatment is two days, once a day, with an interval of 24 h. 16 years old and above take 2 tablets each time, 11–15 years old take 1.5 tablets each time, and 7–10 years old take 1 tablet each time. ATQ obtained the Chinese New Drug Certificate in 2006 and is protected by patents in 40 countries, including the United States. It has been registered and listed in 22 countries, such as Nigeria and Kenya. In addition, it is listed as the first-line drug for the treatment of malignant malaria by the Chinese Ministry of Health. So far, ATQ has shown good safety and efficacy over a two-day course of treatment [2]. Piperaquine is a 4-chloroquinoline that was used to replace chloroquine in China until the 1970 and 1980 s. It has been reported that, compared with piperaquine phosphate, piperaquine exhibited better tolerance in patients and can help reduce treatment costs and duration [3, 4]. Previously, our team reported a large-scale artemisinin-piperaquine mass drug administration research, including total 85–93% of approximately 322,000 inhabitants of Anjouan Island in the Comoros, Africa [5]. Among participants, approximately 32 (0.04%) nulliparous girls below the age of 18 experienced galactorrhea. Despite the low incidence, this adverse reaction raised our concerns about whether ATQ affects female fertility.

Previous toxicity studies devoted to investigating acute and sub-acute toxicity of ATQ in rats, dogs, and monkeys. Such as the half lethal dose (LD_50_) of ATQ in Kunming (KM) mice was 2802.38 mg/kg [6]; prolonged corrected QT (QTc) interval, hepatocyte, and renal tubular necrosis were observed in beagle dogs after oral administration of ATQ 100 mg/kg for 14 days [7]; ATQ at 78.2 and 156.4 mg/kg dose in rhesus monkeys had toxic effects on body weight, food consumption, body temperature, hematologic, biochemical parameters and histological changes [8]. However, in the reproductive and developmental research of ATQ or its components (ART and PQ), more attention is paid to its safety and efficacy in pregnant women infected with malaria [9–15], and a few animal studies focus on embryotoxicity or developmental toxicity [16–18], information available on its reproductive and endocrine toxicity in women and animals is scarce, making it difficult to assess the potential toxicity risk. For the safer clinical application of ATQ, it is important to study its reproductive and endocrine effects. To address the lack of research in this aspect, the present study was to investigate the potential adverse effects of ATQ and its individual components ART and PQ on the reproductive and endocrine systems in female rats through assessing the estrous cycle, serum hormone concentration, and histopathological changes in reproductive and endocrine organs. The results would fill the research gap in the reproductive and endocrine effects of ATQ and hopefully extend our understanding of its underlying mechanisms in this regard.

## Methods

### Test compound and preparation

ART (Lot No. 130601) was purchased from Tongrentai Pharmaceutical Co., Ltd., Sichuan, China. PQ (Lot No. 130536) and ATQ (Lot No. 20130510) were provided by Artepharm Co., Ltd., Guangdong, China. ATQ, PQ, and ART were ground and then formulated with distilled water to obtain suspensions of different concentrations (ATQ: 2.0, 4.0, and 8.0 mg/mL; PQ: 1.5, 3.0, and 6.0 mg/mL; ART: 0.25, 0.5, and 1.0 mg/mL).

### Animals and maintenance

Specific pathogen-free grade Wistar female rats aged 7–8 weeks were obtained from Beijing Vital River Laboratory Animal Technology Co., Ltd., China. The animals were allowed to acclimatize for 5 days before the start of the study. Rats were maintained in an environmentally controlled room under standard laboratory conditions of room temperature (22–25 °C) and relative humidity (43–65%) with a 12 h light/dark cycle. The certified commercial feed diet (Beijing Keaoxieli Feed Co., Ltd., China) and drinking water were available ad libitum. All animal experiments were conducted in compliance with the Principles of Good Laboratory Practice (GLP) from the National Medical Products Administration (NMPA), China, and were performed in the laboratory animal room of the New South Center of Safety Evaluation for Drugs of Guangzhou University of Chinese Medicine, China (Chinese animal use license number: SYXK (Guangdong) 2018-0014). The research protocol was approved by the Institutional Animal Care and Use Committee (IACUC) for animal care and use based on the 3R principle (Reduction, Replacement, and Refinement).

### Dose selection rationale

The dose design of ATQ was based on the results of a pilot study in female rats. The low, medium, and high doses of ATQ in the pilot rat study were 40, 80, and 160 mg/kg, respectively, of which 160 mg/mg was close to the adult clinical equivalent dose based on the body surface area conversion method. After 14 days of administration, female rats in the three dose groups showed obvious reproductive and endocrine toxicity such as abnormal estrous cycle. Because apparent toxicity has already occurred below the therapeutic dose, and we wanted to find out the dose that does not affect the reproductive and endocrine systems, the doses of ATQ in the current study were adjusted down to 20, 40, and 80 mg/kg. According to the component content of ART and PQ in ATQ, the corresponding three doses were designed to be PQ 15, 30, 60 mg/kg and ART 2.5, 5, 10 mg/kg, respectively.

### Experimental design

A total of 160 female rats were randomly divided into ten groups with 16 rats per group: control group (water); ATQ low, medium, and high dose groups (ATQ: 20, 40, and 80 mg/kg); PQ low, medium, and high dose groups (PQ: 15, 30, and 60 mg/kg); ART low, medium, and high dose groups (ART: 2.5, 5, and 10 mg/kg). The control group was designed to monitor whether the experimental conditions were normal, and to compare with the treated groups, etc. The separate PQ groups and ART groups were set to distinguish which component of ATQ plays a role when the animals have adverse reactions, the endocrine effect of a single drug on rats, etc.

The rats in the ATQ, PQ, and ART groups were orally administered with the corresponding dose of ATQ, PQ, and ART, respectively, once a day for 14 days. Distilled water was given to the rats in the control group. The daily application volume (10 mL/kg body weight) of each individual was adjusted once weekly based on body weight. After 14 days of administration, 10 rats from each group were selected to be sacrificed, the remaining rats were stopping administration and recovered for 21 days. Throughout the study, the rats were observed twice daily for clinical signs, such as appearance, behavior, secretions, excretions, respiration, and other toxicity symptoms. Body weight was measured once a week.

After the 5-day acclimation, the estrous cycle of the rats was measured once in the morning and afternoon each day. A wet cotton swab was gently and slowly inserted into the vagina, and the cell sample was removed along the dorsal vaginal wall and then smeared on a glass slide. Papanicolaou was used for staining, and the estrous cycle of the rats was observed under a CX31 biomicroscope (Olympus, Japan) after the smear was dried. The estrous cycle was estimated according to the following criteria. (1) Proestrus: Oval nucleated epithelial cells account for the vast majority, with few white blood cells and keratinized epithelial cells, lasting for 17–21 h. (2) Estrus: Keratinized squamous cells are observed, mostly non-nucleated, while there are cheese-like keratinocytes at the end of estrus, lasting for 9–15 h. (3) Metaestrus: keratinocytes are replaced by small oval nucleated epithelial cells and polymorphonuclear leukocytes, lasting for 14–18 h. (4) Dioestrus: Aged polymorphonuclear leukocytes are seen, along with a small amount of nucleated epithelial cells and mucus, lasting for 48–60 h. The average estrous cycle of female rats is 4–5 days [19]. After two estrous cycles of the rats were observed, the administration was started for 14 days (approximately 3–4 estrous cycles), and the estrous cycle was measured once in the morning and afternoon each day. During the 21-day recovery period (approximately 5–6 estrous cycles), the estrous cycle continued to be observed daily.

Approximately 0.8 mL of blood was collected from all rats with glass capillary at three time points, two days before administration, at the end of administration, and the end of recovery. After being placed overnight at 4–8 °C, the blood samples were centrifuged at 3000 rpm for 10 min at 4 °C, and the separated sera were stored at -80 °C. Serum levels of follicle-stimulating hormone (FSH), luteinizing hormone (LH), prolactin (PRL), estradiol (E2), adrenocorticotrophic hormone (ACTH), and prostaglandin (PG) were determined by enzyme-linked immunosorbent assay (ELISA). These ELISA kits were purchased from Shanghai Yili Biological Technology Co., Ltd, and used the double antibody sandwich method to specifically detect the corresponding hormone concentrations in rat serum and plasma samples. The sensitivities of the ELISA kits to detect FSH, LH, PRL, E2, ACTH, and PG were 0.7 IU/L, 1.5 pg/mL, 10 pg/mL, 2 pg/mL, 2.5 pg/mL, and 15 pg/mL, respectively. The absorbance values of the samples were measured using an Epoch microplate reader (Bio Tek, USA).

At the end of administration and recovery, the rats were subjected to 12–16 h fasting, anesthetized with pentobarbital sodium (30 mg/kg, iv), and euthanized by acute hemorrhage. A complete necropsy was performed on all rats, focusing on the reproductive and endocrine related organs. The weights of the kidney, adrenal gland, uterus, and ovaries were measured. The adrenal gland, uterus, ovaries, and mammary gland were fixed in 10% neutral buffered formalin. These tissues were trimmed, embedded in paraffin, sectioned, stained with hematoxylin and eosin, and then evaluated by an experienced pathologist using a DMLB biomicroscope (Leica, Germany).

### Statistical analysis

The data were presented as mean and standard deviation (mean ± SD). The statistical analyses were performed using SPSS 19.0 software. If the variance was homogeneous, the difference between groups was evaluated by the one-way analysis of variance (ANOVA) test. If not, the Kruskal-Wallis non-parametric test was used. If either of the tests showed a significant difference between groups, the analysis was continued by the multiple comparison procedure of Dunnett’s test. Differences were considered statistically significant when *P* ≤ 0.05.

## Results

### Clinical sign and body weight

There was no abnormality in the autonomic activities (defecation, urination, breathing, pupillary reflex, salivation, grooming, etc.), sensory function (touch response, auditory reflex, tail clamping reflex, grasping reflex, etc.), and neuromuscular function (gait, movement, etc.) of the rats in each group. The rats did not have central nervous system toxicity such as convulsion and twitching. Statistical evaluation of the body weights showed no significant difference among groups at all time points during the study.

### Estrous cycle

The estrous cycle of all rats before administration was regular and complete, with an average estrous cycle of 4.6 ± 0.8 days. During the administration and recovery period, there was no obvious abnormality in the estrous cycle of rats in the control group and the three dose groups of ART, and each estrous cycle was maintained within 4–5 days. However, the rats in all ATQ and PQ dose groups showed estrous cycle disorder within 4–7 days after administration, and the time in metaestrus was significantly prolonged. Then no estrous manifestation was observed after 7 days of administration, and the estrous cycle maintained in metaestrus to dioestrus. Until the end of the 21-day recovery period, there was still no estrus.

### Endocrine hormone level

**Fig. 1:**
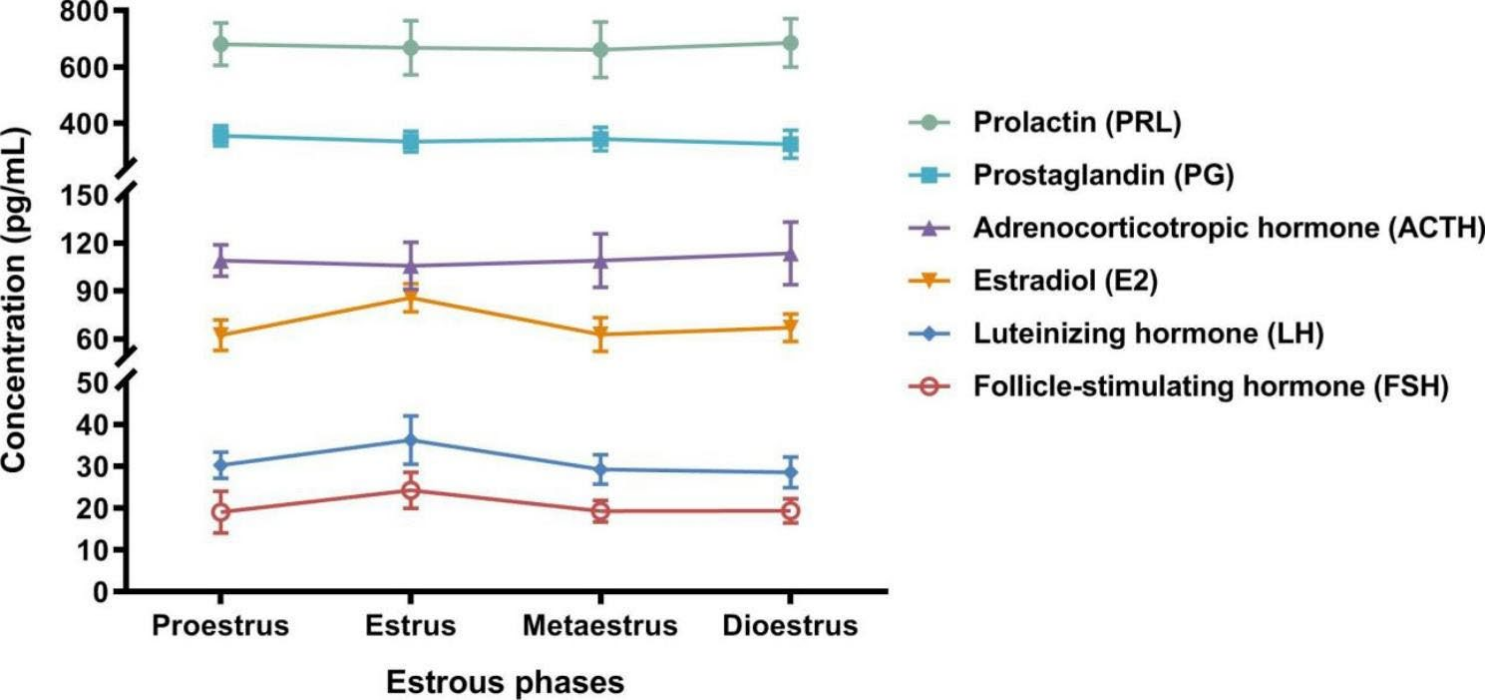
The endocrine hormone levels of the female rats in an estrous cycle before administration, including proestrus (n = 24), estrus (n = 23), metaestrus (n = 29), and dioestrus (n = 84). The tested hormones are follicle-stimulating hormone (FSH), luteinizing hormone (LH), prolactin (PRL), estradiol (E2), adrenocorticotrophic hormone (ACTH), and prostaglandin (PG). Except that the unit of FSH is IU/L, the other hormones are pg/mL

Two days before administration, the animals were not yet grouped. The hormonal levels were measured in 160 rats, of which 24, 23, 29, and 84 rats were in proestrus, estrus, metaestrus, and dioestrus, respectively. As shown in Fig. 1, PRL, PG, and ACTH in rats before administration did not change significantly during a complete estrous cycle. Although E2, LH, and FSH were slightly elevated during estrus, there was no significant difference with other estrous phases. This result suggests that the different phases of the estrous cycle in female rats have no obvious effect on the measured hormones.

At the end of administration, only 3, 1, 1, and 2 rats were found in estrus in the control group and ART low, medium, and high dose group, respectively, all hormonal values of these rats did not change significantly. Therefore, even though the rats in each group were not all in the same estrous phase, the endocrine hormones were expressed as overall levels.

The endocrine hormonal values of female rats in each group at the end of administration are summarized in Table 1. The oral administration of ATQ, PQ, or ART at three doses did not significantly change the serum levels of E2, FSH, and LH in rats as compared to the control group. Some significant decreases of PRL were observed in rats after administration of ATQ at three doses, which were not considered toxicologically meaningful since the decreases were slight and within historical values of rats before administration. Compared with the control group, ATQ and PQ significantly reduced PG and increased ACTH in rats at three doses, and showed a dose-dependent relationship. Although there were no statistically significant differences in hormones measured at the end of recovery, the ACTH level of rats in all PQ dose groups was slightly higher compared with the control group.

**Table 1:** The endocrine hormonal levels of the female rats after administered with ATQ, PQ, or ART at three doses for 14 days (n = 16)

Group	E2 (pg/mL)	FSH (IU/L)	LH (pg/mL)	PRL (pg/mL)	PG (pg/mL)	ACTH (pg/mL)
Control	60.63 ± 12.18	22.38 ± 3.79	33.19 ± 6.06	729.7 ± 79.8	369.6 ± 25.2	108.4 ± 16.0
ART low-dose	64.15 ± 7.54	20.71 ± 4.27	33.82 ± 4.62	746.3 ± 63.5	345.8 ± 57.2	111.3 ± 18.1
ART mid-dose	65.07 ± 4.53	21.40 ± 3.59	35.11 ± 4.26	740.6 ± 47.9	352.9 ± 27.5	108.1 ± 14.5
ART high-dose	65.65 ± 4.84	21.08 ± 2.48	34.55 ± 5.24	710.1 ± 68.5	357.6 ± 36.0	115.5 ± 17.7
PQ low-dose	63.13 ± 9.56	20.28 ± 1.79	29.53 ± 6.87	660.2 ± 50.1	323.4 ± 44.1^*^	129.0 ± 13.5^*^
PQ mid-dose	62.14 ± 6.76	23.04 ± 2.75	31.98 ± 7.61	674.9 ± 85.6	319.2 ± 41.5^*^	127.3 ± 8.4^*^
PQ high-dose	70.81 ± 11.87	22.12 ± 2.81	29.73 ± 7.27	708.1 ± 68.3	313.3 ± 23.3^*^	132.6 ± 16.4^*^
ATQ low-dose	55.46 ± 11.46	21.31 ± 2.43	32.93 ± 11.75	634.4 ± 46.8^*^	308.6 ± 52.8^*^	131.8 ± 35.9^*^
ATQ mid-dose	61.66 ± 9.57	21.35 ± 3.32	33.24 ± 12.89	637.3 ± 91.8^*^	302.1 ± 29.0^*^	129.5 ± 16.4^*^
ATQ high-dose	54.19 ± 10.22	19.30 ± 3.47	29.34 ± 5.26	595.8 ± 51.9^*^	293.9 ± 60.8^*^	141.0 ± 17.1^*^

^*^ Significantly different from the control group at *P* < 0.05. One-way ANOVA test was performed, followed by Dunnett’s test

### Organ weight and histopathology

The weights of the kidney, adrenal gland, ovary, and uterus of rats at the end of administration are presented in Table 2. The weights of the ovary and uterus of rats in all treated groups did not change significantly. However, through gross anatomy, it was found that at the end of administration, the uterus of rats in all ATQ and PQ dose groups did not show relevant changes caused by estrus, such as congestion, swelling, and uterine fluid filling, which was consistent with the results of estrous cycle determination (i.e. the rats in a persistent dioestrus phase). The kidney and adrenal gland of rats in all ATQ and PQ dose groups increased in weight and size at the end of administration, but did not return to completely normal after 21 days of recovery.

**Fig. 2:**
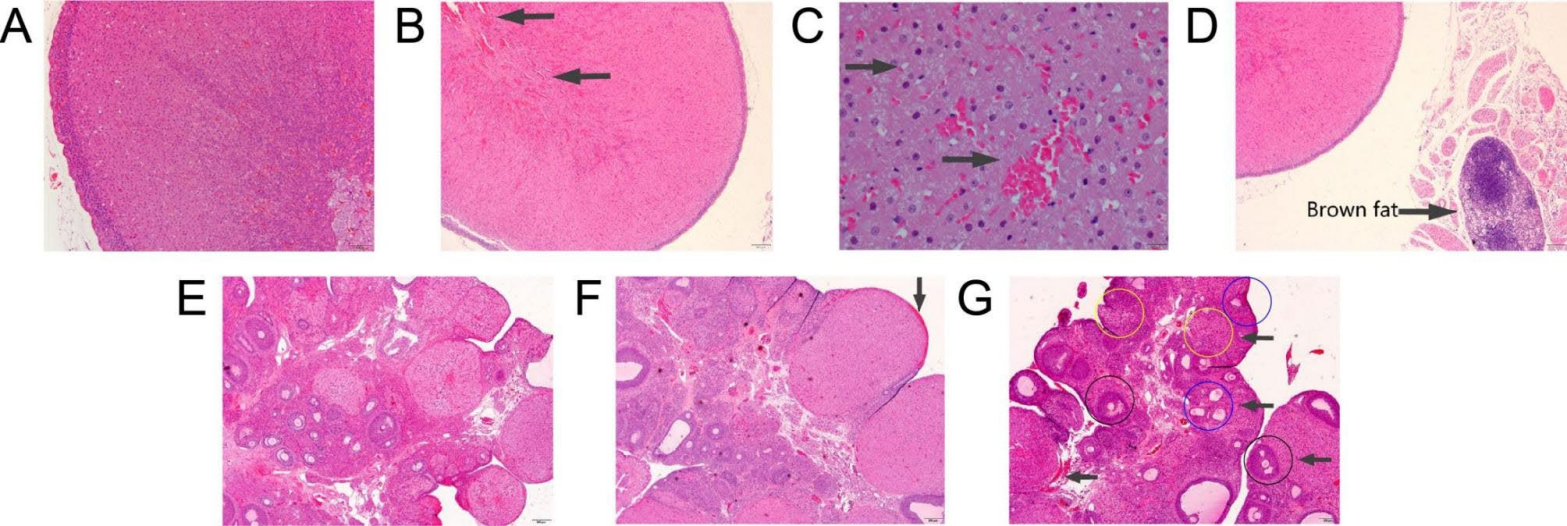
The representative histological photomicrographs of the adrenal gland (top row) and ovary (bottom row) of female rats after administered with ATQ for 14 days. (A) Control adrenal gland (×100); (B) Adrenal gland of 80 mg/kg ATQ (×40). Vasodilation and hyperemia in adrenal cortex and medulla; (C) Adrenal gland of 80 mg/kg ATQ (×400). Vacuolar degeneration and hyperemia; (D) Adrenal gland of 80 mg/kg ATQ (×40). Brown fat attached to the adrenal gland; (E) Control ovary (×40); (F) Ovary of 80 mg/kg ATQ (×40). Hyperemia in the luteal surface; (G) Ovary of 80 mg/kg ATQ (×40). Numerous but small corpus luteum, and disordered follicle development (mainly primary and secondary follicles, few or no mature follicles). Yellow, blue, and black circles are representative of the corpus luteum, primary follicle, and secondary follicle, respectively. Arrows indicate histopathological changes

After 14 days of administration, histopathological examination showed no significant changes in the uterus and mammary gland of the treated rats, while the adrenal gland and ovary appeared pathological lesions as illustrated in Fig. 2. The adrenal gland and ovary of rats in all ATQ and PQ dose groups were pathologically damaged. In the adrenal gland, vasodilation and hyperemia in the cortex and medulla were observed, as well as hyperplasia and vacuolar degeneration. In the ovary, the lesions included corpus luteum surface hyperemia, numerous but small corpus luteum, and disordered follicle development (mainly primary and secondary follicles, few or no mature follicles). Additionally, during gross anatomy, the rats in the above groups were found to have a large amount of fat attached to the adrenal gland and adhered to the stomach and intestines, which were revealed to be brown fat through microscopic examination (Fig. 2D). After 21 days of drug withdrawal, the above pathological lesions were improved, but not completely recovered.

**Table 2:** The organ weights of the female rats after administered with ATQ, PQ, or ART at three doses for 14 days (n = 10)

Group	Kidney (g)		Adrenal gland (g)		Ovary (g)		Uterus (g)
	Left	Right	Left	Right	Left	Right	
Control	0.898 ± 0.075	0.908 ± 0.054	0.052 ± 0.006	0.049 ± 0.005	0.067 ± 0.011	0.074 ± 0.011	0.503 ± 0.218
ART low-dose	0.860 ± 0.059	0.883 ± 0.066	0.051 ± 0.006	0.049 ± 0.004	0.072 ± 0.026	0.065 ± 0.015	0.488 ± 0.172
ART mid-dose	0.887 ± 0.085	0.940 ± 0.095	0.052 ± 0.006	0.046 ± 0.004	0.065 ± 0.012	0.065 ± 0.017	0.468 ± 0.180
ART high-dose	0.851 ± 0.064	0.877 ± 0.068	0.056 ± 0.012	0.052 ± 0.017	0.068 ± 0.017	0.070 ± 0.014	0.607 ± 0.200
PQ low-dose	1.062 ± 0.054^*^	1.094 ± 0.037^*^	0.068 ± 0.011^*^	0.059 ± 0.004^*^	0.068 ± 0.016	0.069 ± 0.010	0.461 ± 0.145
PQ mid-dose	1.064 ± 0.099^*^	1.114 ± 0.082^*^	0.068 ± 0.009^*^	0.062 ± 0.008^*^	0.068 ± 0.011	0.067 ± 0.008	0.444 ± 0.110
PQ high-dose	1.052 ± 0.087^*^	1.097 ± 0.104^*^	0.072 ± 0.012^*^	0.066 ± 0.011^*^	0.067 ± 0.013	0.069 ± 0.012	0.427 ± 0.125
ATQ low-dose	1.137 ± 0.135^*^	1.182 ± 0.171^*^	0.069 ± 0.008^*^	0.062 ± 0.009^*^	0.072 ± 0.024	0.074 ± 0.018	0.516 ± 0.258
ATQ mid-dose	1.195 ± 0.140^*^	1.267 ± 0.146^*^	0.070 ± 0.012^*^	0.063 ± 0.011^*^	0.068 ± 0.019	0.067 ± 0.014	0.473 ± 0.089
ATQ high-dose	1.193 ± 0.114^*^	1.247 ± 0.156^*^	0.074 ± 0.010^*^	0.065 ± 0.009^*^	0.071 ± 0.016	0.079 ± 0.012	0.446 ± 0.077

^*^ Significantly different from the control group at *P* < 0.05. One-way ANOVA test was performed, followed by Dunnett’s test

## Discussion

The experimental results showed that ART has no significant effect on reproductive and endocrine systems in female rats, since all the examined items were normal, including general observation, estrous cycle, hormonal level, organ weight, and histopathological examination. The female rats in all ATQ and PQ dose groups had no estrus, decreased PG, increased ACTH, increased weights of the kidney and adrenal gland, and pathological lesions of the adrenal gland and ovary. Therefore, most of the reproductive and endocrine effects of ATQ in female rats may come from PQ. The current results of ART were similar to those of a previous reproductive toxicity study in rats [20], which showed that ART with oral doses of 7, 35, and 70 mg/kg for 7 days did neither cause changes in LH, PRL, and estrogen levels nor pathological lesions in ovaries and uterus, except for the decrease in FSH and increase in progesterone possibly caused by neurotoxicity.

In the current study, the increases in adrenal weight and size of rats in ATQ and PQ groups were mainly due to the hyperplasia of the adrenal cortex and medulla and its vasodilation and hyperemia. Besides, the production of ACTH in the rats of these groups was promoted. Therefore, it is suggested that ATQ and PQ could induce adrenal hyperplasia in rats, which is in correspondence with the results of our previous repeated dose toxicity studies in rats and monkeys. Since adrenal hyperplasia can lead to blood electrolyte disorder, the serum biochemical results showed that potassium and chloride decreased significantly after oral administration of ATQ at doses of 63, 126, and 252 mg/kg in rats for 28 days (unpublished data), while a significant decrease in serum sodium and hypertrophy of adrenal cortical parenchymal cells were observed after oral administration of ATQ at doses of 39.1, 78.2, and 156.4 mg/kg in monkeys for 21 days [8].

Adipose organs are mainly composed of white adipose tissue and brown adipose tissue. The color distinction between a “brown” and a “white” adipocyte largely reflects the many more mitochondria (which are high in iron) in brown adipocytes compared to white adipocytes. White adipose tissue stores energy in the form of triglycerides and secretes hormones and cytokines that affect energy balance, while brown adipose tissue consumes energy through the coupling of lipid oxygen in mitochondria to produce heat. The function of brown fat to produce heat is the result of the specific expression of uncoupling protein 1 (UCP1). The brown fat exists within small mammals in distinct locations being innervated by the sympathetic nervous system (SNS). During cold stress, brown fat thermogenesis is classically stimulated by norepinephrine released from SNS, which activates β3-adrenergic receptors in brown adipocytes [21–23]. Seale et al. pointed out that brown fat cells emerge in white adipose tissue in response to prolonged β-adrenergic stimulation [24]. The present study showed that the rats in ATQ and PQ groups had a large amount of brown fat attached around the adrenal glands, possibly because adrenal hyperplasia leads to abnormal secretion of norepinephrine and epinephrine, which in turn affects fat metabolism.

Previous studies have shown that quinolines cause estrous cycle disorders and ovulation abnormalities in animals. Oral administration of chloroquine for four weeks was found to alter the estrous cycle in rats (i.e. the rats showed a persistent dioestrus smear), lower serum estrogen and LH levels, while serum FSH was unaltered [25]. Gavage of amodiaquine hydrochloride in rats for 28 days significantly increased the diestrus phase and reduced the number of ova shed on estrus, but there was no significant difference in the serum concentrations of FSH, LH, and PRL [26]. Quinine administered orally for 28 days completely blocks ovulation, suppresses LH surge, and produces oxidative stress in the ovary [27]. As a tetraaminoquinoline drug, the experimental results of PQ in our study were basically in line with the finding of these previous studies: the estrous cycle of rats was disrupted within 4–7 days after PQ or ATQ administration, and then led to a persistent dioestrus phase. But endocrine hormonal levels were not significantly affected, including E2, FSH, PRL, and LH. The ovaries of these rats had pathological lesions, including corpus luteum surface hyperemia, numerous but small corpus luteum, and disordered follicle development. The significant decrease in serum PG is likely due to the antagonistic effect of PQ, as the antimalarials like chloroquine can stabilize lysosomes and are PG antagonists [28]. PQ-induced abnormal follicular development may also be associated with decreased PG. Decreased PG would slow luteolysis, which may be one of the reasons for the numerous but small corpus luteum. Although PQ had no pathological damage to the uterus in this study, it is not excluded that PQ does not affect the uterus since PG has an effect on the contractility of the smooth muscles of the uterus. As reported, chloroquine inhibits the uterine contraction process in animals [29, 30].

Unfortunately, progesterone was not detected in this study, which is important in determining the functional status of the corpus luteum. Pathological examination results showed that the corpus luteum of female rats in ATQ and PQ groups were congested and smaller, suggesting the function of corpus luteum might be impaired, leading to the change of progesterone. Thus, progesterone is recommended to be included in monitoring in the subsequent clinical application of ATQ.

Before the study, we supposed that after 14 days of dosing, rats might experience some responses similar to or related to galactorrhea of young nulliparous. However, there was no lactation, pathological damage to the mammary gland, or increased PRL in the treated female rats. Of note, the clinical incidence of ATQ-induced galactorrhea in girls is low (0.04%), the number of animals in our experiment is small, and there are species differences between rats and humans. So these may be the reasons for the absence of lactation-related endocrine changes in the treated rats. In addition, the serum levels of E2, FSH, and LH were also unaltered, but it cannot be excluded that these hormones are not affected after the drug treatment in humans. Therefore, when ATQ or PQ-containing drugs are used in special populations or reproductive endocrine-related adverse reactions occur after treatment (such as galactorrhea in nulliparous girls), it is recommended to monitor these hormones to fully understand the endocrine status in patients.

## Conclusion

According to the results presented in this study, ART had no obvious reproductive and endocrine effects on female rats, while ATQ and PQ caused adrenal hyperplasia, increased ACTH, decreased PG, blocked estrus, corpus luteum surface hyperemia, and disrupted follicle development in female rats. These events suggest that ATQ and PQ may interfere with the female reproductive and endocrine systems, potentially reducing fertility.

## Data Availability

The original contributions presented in the study are included in the article/Supplementary Material, further inquiries can be directed to the corresponding authors.

## Abbreviations

ACTH: Adrenocorticotropic hormone
ART: Artemisinin
ACT: Artemisinin-based combination therapy
ATQ: Artemisinin-piperaquine tablets
E2: Estradiol
FSH: Follicle-stimulating hormone
LH: Luteinizing hormone
PQ: Piperaquine
PRL: Prolactin
PG: Prostaglandin
WHO: World Health Organization
